# Multiple ionization of iodine for 2.5–5.0 MeV I^22+^ ions impacting on Fe target

**DOI:** 10.1038/s41598-022-10337-2

**Published:** 2022-04-15

**Authors:** Xianming Zhou, Jing Wei, Rui Cheng, Changhui Liang, Yanhong Chen, Xiaoan Zhang, Yongtao Zhao

**Affiliations:** 1 Ion Beam and Optical Physics Joint Laboratory of Xianyang Normal University and IMP, CAS, Xianyang Normal University, Wenlin Rd. 01, XianyangShannxi, 712000 China; 2 Institute of Modern Physics, Chinese Academy of Sciences, Lanzhou, 730000 China; 3 School of Science, Xi’an Jiaotong University, Xi’an, 710049 China

**Keywords:** Atomic and molecular collision processes, Electronic structure of atoms and molecules

## Abstract

The L-shell X-ray emissions of iodine are investigated as a function of the incident energy for I^22+^ ions impacting on Fe target in the energy region near the Bohr velocity. Six distinct L-subshell X-rays, Lι, Lα_1, 2_, Lβ_1, 3, 4_, Lβ_2, 15_, Lγ_1_ and Lγ_2, 3, 4, 4_^'^, are observed. Compared to the atomic data, the energy of the experimental X ray shifts to the higher energy side. The relative intensity ratios of Lι, Lβ_1, 3, 4_, Lβ_2, 15_, to Lα_1, 2_, Lι to Lβ_2, 15_ and Lγ_2, 3, 4, 4/_ to Lγ_1_ are enhanced, but has no obvious change with the increase of projectile energy in the present energy region. That is interpreted by the multiple ionization effect of the M-, N- and O-shell electrons.

## Introduction

The study of inner-shell ionization induced by highly charged ion-atom collisions can not only provide fundamental data for theoretical simulation of atomic-molecular reaction dynamics and analysis of cosmic astrophysics, but also have important practical applications in material modification, elemental analysis, warm and dense plasma diagnostics and so on^1–5^. Since the 1950s, due to the development of accelerator technology and the advancement of detection and analysis method, the related research has received extensive attention and made great achievements^6–48^. During the interaction of highly charged heavy ion with solids, above the target surface, the incident ions can capture the valance electrons of the surface target atoms to the high Rydberg state trough resonant capture to form the first hollow atoms (HA1)^22–24^. Then, it can enter the lower surface to interact with the target atoms at close range, and capture electrons from the inner-shell of the target atom to the shell with a smaller main quantum number in the way of side feeding, and form a more compact hollow atom (the second hollow atom, HA2) to achieve the neutralization^25,26^. In addition, the inner shell electrons of target atoms and projectile ions can be ionized by Coulomb collisions. The de-excitation of these excited atoms can emit X rays in the form of radiation, or excite Auger electrons in the form of radiationless Auger transition and CK (Coster-Kornig) transition^27,28^. The energy and broadening of characteristic X ray reflect the energy level structure and electron distribution of excited atoms. The X-ray production cross section can give the information of ionization probability of inner shell electrons^29–32^. So, X-ray emission measurement is an important method to experimentally study the atomic characteristics and the mechanism of atomic inner-shell ionization process in ion-atom collisions.

Previous studies on the interaction of highly charged ions with atoms can be divided into two categories. One is in the low energy region below the Bohr velocity. Experimentally, taking the research of Briand et al. as an example^33–36^, using a crystal spectrometer, the formation mechanism of hollow atoms near the target surface was expounded through the high resolution structure analysis of K-shell X-ray. In theory, Burgdörfer established a classical over-the-barrier model (COBM) to describe this process^37^. The other is concentrated in the energy range of tens to hundreds MeV. Through the experimental measurement and theoretical analysis of X-ray emission cross section, the ionization of the inner shell is studied. And many well-established theories are developed to simulate such process, such as, Binary Encounter Approximation (BEA), Plane Wave Born Approximation (PWBA) and ECPSSR model (PWBA modified by Energy-loss, Coulomb-deflection, and Perturbed-Stationary-State Relativistic)^38–40^. However, in the energy region near the Bohr velocity, due to the limitation of experimental conditions, the relevant experimental research is relatively rare, and the theoretical description is still inconclusive.

Different from the neutralization de-excitation of the first and second generation hollow atoms formed by low velocity ions near the solid surfaces, the inner shell process generated by the impact of highly charged ions near the Bohr velocity has its own uniqueness. In addition to the neutralization process of electron capture, the projectile has enough energy to enter the target and interact with the target atoms in a close distance, and that result in Coulomb ionization. With the de-excitation of the holes in the inner shell, the outer shell may be in a multiple ionization state. That causes an energy shift of the X ray and a change in the relative intensity ratio of the sub-shell X ray. In our previous work, such multiple ionization of projectile has been observed and the effect of charge state and target atomic number has been verified^16,41^. Here, we would like to present the further research, and the special attention will be devoted to the influence of incident energy on the multiple ionization.

In this work, the highly charge heavy ions of I^22+^ with energy from 2.5 to 5.0 MeV (the velocity is about 0.89 to 1.26 times *v*_0_, *v*_0_ = 2.19 × 10^6^ m/s is the Bohr velocity) were used to bombard the solid Fe target. The L-subshell X rays were detected. The emission, energy shift and relative intensity ratio of the X rays were analyzed. The formation of multiple ionization states of the projectile and its effect on the X-ray emission were discussed.

## Experimental method

**Figure 1:**
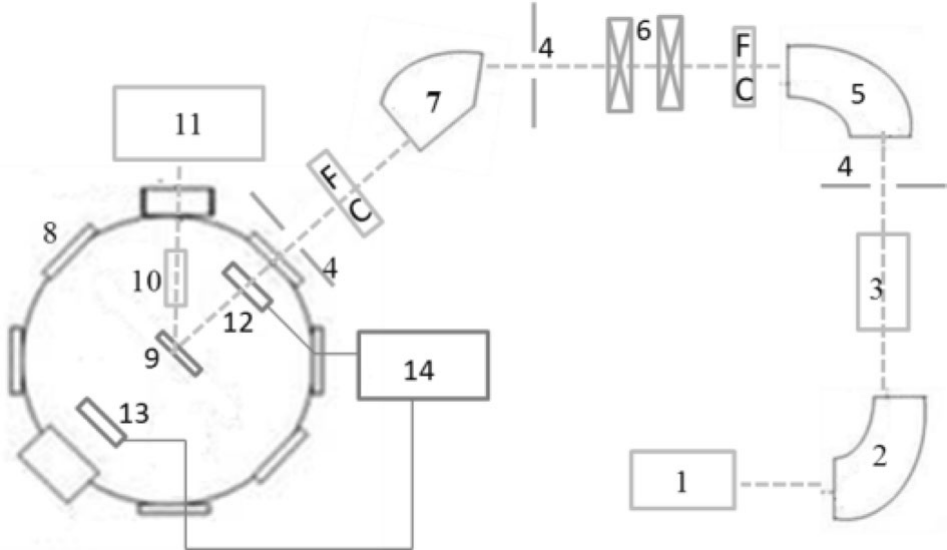
Schematic drawing of experiment setup: 1, ECR ion source, 2, analyzing magnet, 3, high volt accelerate platform, 4, barrier, 5, 90° deflection magnet, 6, magnetic quadrupled lens, 7, 60^◦^ deflection magnet, 8, ultrahigh vacuum target chamber, 9, target, 10, silicon drift detector, 11, X-ray recording system, 12, penetrable faraday cup, 13, common faraday cup, 14, projectile number recording system.

The measurements have been carried out at the 320 kV high voltage experimental platform at the Institute of Modern Physics, Chinese Academy of Sciences (IMP, CAS) in Lanzhou, China. More details of the experimental system have been described in a previous work^42^. The experimental setup is shown in Fig. 1. In brief, the I^22+^ ions are produced and extracted from the Electron Cyclotron Resonance (ECR) ion source and selected by a 90° analyzing magnet, and then introduced into the ultrahigh vacuum target chamber (10^–8^ mbar) after acceleration, focus, multi-deflections and multi-collimations. The divergence of the beam is smaller than 0.2°. The ion beam impacts perpendicularly onto the target with a spot size of about Φ 3 mm. The target, prepared carefully in the laboratory, having a purity of 99.99% with surface area of 15 × 20 mm^2^ and a thickness of 0.1 mm, is positioned on a sample holder. It permitted a three-dimensional movement to change freely the target position and remove away from the beam line in order to measure the current.

The emitted X-rays are detected by a Silicon Drift Detector (SDD) produced by AMPTEK. The SDD has an effective detection area of 7 mm^2^ and a 12.5 μm Be window in the front of the detector. The SDD is placed at 80 mm far away from the target surface in the chamber and at 135^◦^ angle with respect to the beam direction. The detector has an effective energy range of 0.5–14.3 keV when the gain was selected at 100, and an energy resolution of about 136 eV at 5.9 keV. The energy calibration is performed using simultaneously two standard radioactive sources of ^55^Fe and ^241^Am, and then tested by measuring the energies of the K-shell X ray of Al, V and Fe produced by photon irradiation. In this way, a precise measurement of the X-ray energy can be guaranteed. The SDD intrinsic efficiency, which combines the effects of transmission through the Be window and the interaction in the silicon detector, is well determined by transmission measurement.

The number of incident projectiles, which could not be measured immediately by recording the target current due to the influence of the secondary electron emission, is detected indirectly by the combined use of a penetrable Faraday cup and a common one. Before every measurement, the projectile number detected by the penetrable Faraday cup (N_1_) and the common Faraday cup (N_2_) were recorded. The ration (R) of N_2_/N_1_ was checked. During the X-ray detection, the projectile number detected by the penetrable Faraday cup (N_1_′) was recorded. Therefore, the number of the projectile impacting on the target (N) could be calculated by the formula of N = N_1_′*R.

## Results and discussion

### L-shell X-ray emission of iodine

**Figure 2:**
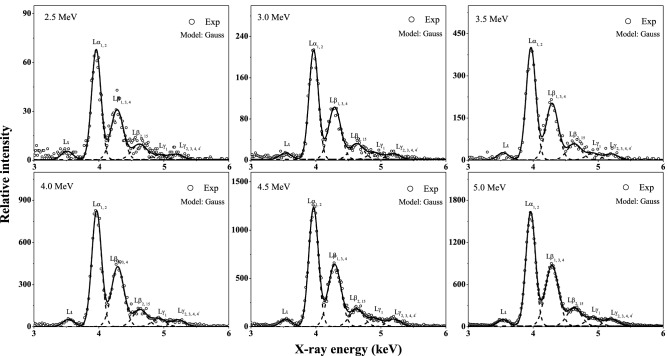
Characteristic X-ray spectra of I for I^22+^ ions with various incident energy impacting on Fe target.

In Fig. 2, the typical X-ray emission spectra of I for I^22+^ ions impacting on Fe target are presented as a function of incident energy. The spectra are normalized by the projectile number and well fitted by a nonlinear curve Gaussian fitting program. The structures of the spectra are similar for projectile with different energy. It consists mainly of six distinct L-subshell lines and can be identified as Lι, Lα_1, 2_, Lβ_1, 3, 4_, Lβ_2, 15_, Lγ_1_ and Lγ_2, 3, 4, 4_^'^ X ray, which results from the radiation transition of M_1_–L_3_, M_5, 4_–L_3_, M_4_–L_2_/M_3, 2_–L_1_, N_5, 4_–L_3_, N_4_–L_2_ and N_3, 2_–L_1_/O_3, 2_–L_1_, respectively^43,44^.

In the present work, the velocity of 2.5–5.0 MeV I^22+^ ions is about (1.94–2.75) × 10^6^ m/s. The distance between the ion source and the target surface is about 12.74 m. Taking into account the movement before acceleration, the minimum flight time of the incident ions to the target surface is also greater than 3.62 × 10^–6^ s. This is long enough for any decay of the projectile’s original metastable. So, the present results can be eliminated for the decay of metastable state above the target surface. The initial electronic configuration of the projectile I^22+^ ion is [Ar] 3d^10^4s^2^4p^1^. There is no initial L-shell vacancy. Therefore, the experimental spectrum is not the result of the de-excitation of the first generation hollow atoms on the upper surface. However, the corresponding holes and upper-level electrons are prerequisite for L-shell X-ray emission. The present experimental result indicates that, along with the neutralization process of electron capture, the inner-shell electrons of the incident ion are also ionized by collisions with the target atom within the target, and the second hollow atoms are formed. In the present work, the L-subshell X rays of I mainly come from the de-excitation of the second hollow atom below the surface.

### Multiple ionization state of iodine ions

The measured energies of the six I L-subshell X ray are tabulated in Table 1. There are no obvious regular changes for different incident energy, and it is almost a constant within the estimated error, but larger than the value of a singly ionized atom^43,44^. For example, an average blue shift of 51±5 eV, 26±3 eV, 60±5 eV, 117±5 eV, 110±7 eV and 134±9 eV for Lι, Lα_1, 2_, Lβ_1, 3, 4_, Lβ_2, 15_, Lγ_1_ and Lγ_2,3,4,4′_ X ray is observed, respectively. According to the discussion of section ‘L-shell X-ray emission of iodine’, the L X-ray emission of I occurs after the collisions below the surface, where the projectile has been slowed down due to the energy loss of collision, even though the initial velocity of I^22+^ ions is larger. Therefore, the effect of Doppler shift on the line shift can be neglected. It is proposed that the observed blue shift arise primarily from the multiple ionization of outer-shell electrons, such as M-, N- and O-shell electrons.

**Table 1:** The energies of I L-subshell X-rays produced by I^22+^ ions with various incident energies impacting on Fe target.

Incident energy (MeV)	Lι (eV) ± 5 eV	Lα_1,2_ (eV) ± 3 eV	Lβ_1, 3, 4_ (eV) ± 5 Ev	Lβ_2, 15_ (eV) ± 5 eV	Lγ_1_ (eV) ± 7 eV	Lγ_2,3,4,4_' (eV) ± 9 eV
2.5	3535	3960	4284	4625	4913	5190
3.0	3534	3959	4286	4622	4909	5192
3.5	3538	3965	4287	4626	4912	5195
4.0	3536	3966	4289	4625	4916	5207
4.5	3538	3965	4289	4627	4907	5201
5.0	3537	3964	4287	4623	4916	5208
Average	3536	3963	4287	4625	4912	5199
Atomic^43,44^	3485	3937	4227	4508	4802	5065
Shift	51	26	60	117	110	134

During the collision of highly charged heavy ions with atoms, in addition to the ionization of inner-shell electrons, the multiple ionization state may be generated owing to the dual results of Coulomb ionization and electron capture. In this case, the screening of the nuclear is reduced because of the absence of outer-shell electrons, and the binding energies of the remaining orbital electrons are perturbed. As a result, the corresponding X-ray energy is enlarged, namely, the experimental blue shift is obtained. For example, the radiation energy of the M_5_-L_3_ transition for I atom is about 3939 eV, but that is about 3976 eV for I^22+^ ions, which is an increase of about 37 eV over the atomic data. So, it is proposed that, in the present work, in the interaction of the I^22+^ ions with the target atom, in addition to the single ionization of L-shell electrons, under the synergistic effect of ionization and capture, the M-, N- and O-shells form multiple ionization states which are different from the initial electron configuration.

If leaving the subsequent excitation by non-radiative transitions and the effect of electron correlation out of account, the multi-ionization cross section can be written in the form of the product of the single ionization cross section^45^. Multiple ionization degree is proportional to that of single ionization. Single ionization can be produced by Coulomb ionization or charger transfer. According to the estimation of PWBA theory and Oppenheimer–Brinkman–Kramer (OBK) approximation^46^, the single ionization cross section of I L-shell produced by I^22+^ ions impacting on Fe target is in the order of 1 barn, and that is about 10^5–6^ barn for the M-shell electrons. The cross section increases with the increase of the incident energy, but the increase is not large, in the present experimental energy region. For example, the ionization cross section of M_4,5_ electrons at the incident energy of 5 MeV is about 9 times larger than that at 2.5 MeV. This value is only about 3 and 6 for M_2, 3_ and M_1_ electrons, respectively. Therefore, there should be no obvious change in the multi-ionization degree of the outer-shells with the incident energy. This can also be verified from the change in the relative intensity ratio of the sub-shell X rays, as discussed below. As a result, no obvious change in the blue shift of the experimental spectra line was observed with the incident energy, as shown in Table 1.

### Influence of multiple ionization on the relative intensity ratios of the L-subshell X rays

**Figure 3:**
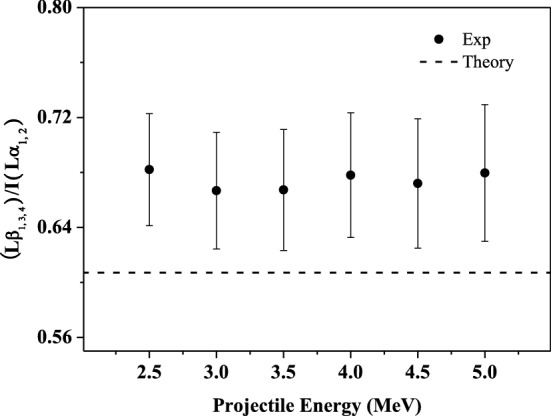
Relative intensity ratios of I Lβ_1, 3, 4_ to Lα_1, 2_ X-ray as a function of incident energy.

**Figure 4:**
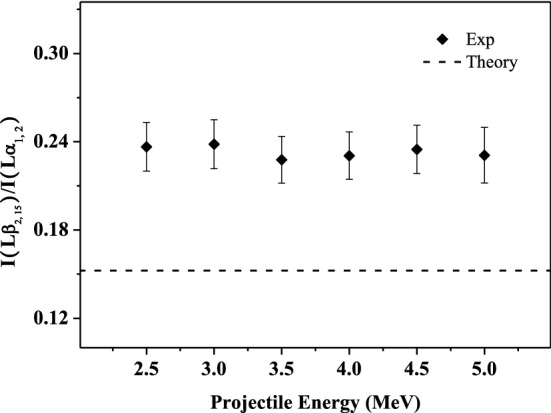
Relative intensity ratios of I Lβ_2, 15_ to Lα_1, 2_ X-ray as a function of incident energy.

**Figure 5:**
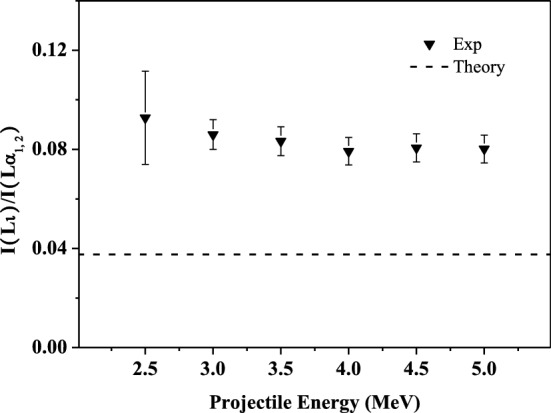
Relative intensity ratios of I Lι to Lα_1, 2_ X-ray as a function of incident energy.

**Figure 6:**
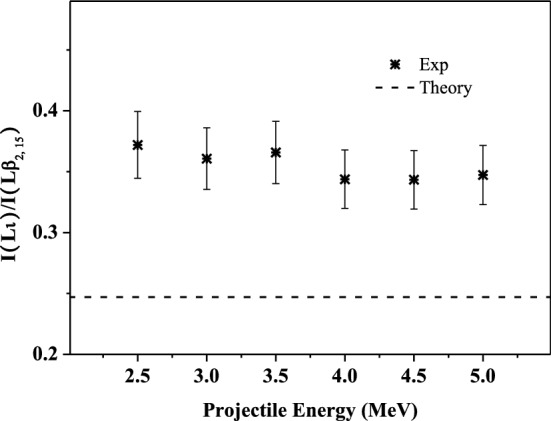
Relative intensity ratios of I Lι to Lβ_2, 15_ X-ray as a function of incident energy.

**Figure 7:**
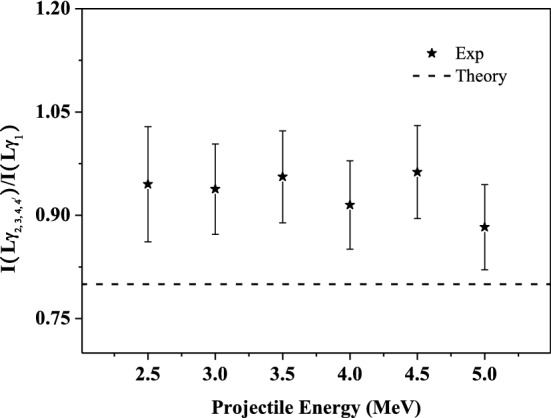
Relative intensity ratios of I Lγ_2, 3, 4, 4_' to Lγ_1_ X-ray as a function of incident energy.

The multiple ionization not only causes the blue shift of the X-ray energy, but also affects the fluorescence yield of each subshell X rays. This will result in a change in the relative intensity ratio of the observed spectral lines. Figures 3, 4, 5, 6 and 7 show the relative intensity ratios of the L-subshell X ray as a function of the incident energy. The experimental error mainly comes from the X-ray count statistics, and the maximum value is about 10%. For comparison, the theoretical atomic data for single ionization are also given, which is obtained from the ECPSSR calculation. It can be found that the experimental values are larger than the theoretical atomic data and almost identical at different incident energy within the error. This can be understood in terms of the multiple ionization states of the projectile.

Lβ_1, 3, 4_ X ray mainly contains three lines of the radiation transition from M electrons filling the L_2_- and L_1_-subshell vacancies. For iodine, the theatrical relative intensity ratio of M_4_-L_2_ (corresponding to Lβ_1_ X ray) to M_3, 2_-L_1_ (corresponding to Lβ_3, 4_ X ray) radiation transition is about 10 to 1^47,48^. Lβ_1, 3, 4_ and Lα_1, 2_ X ray can be roughly regard as the main results of transitions of M_4, 5_ electrons to L_3_ and L_2_ vacancies, and the corresponding fluorescence yield is 0.021 and 0.038, respectively. The Auger yield a_2_ and a_3_ for the L_2_ and L_3_ subshell are 0.767 and 0.921^47,48^. They are all in the same order of magnitude, and not much different^47,48^. When the outer shells are in the multiple ionization states, the Auger transition probability of L_2_ and L_3_ vacancies will be reduced in the same proportion, and which will result in an enhancement of the fluorescence yield of the M_4_-L_2_ and M_5, 4_-L_3_ transition by almost the same magnitude. So, this will not cause a significant change in the relative intensity ratio of Lβ_1, 3, 4_ and Lα_1, 2_ X rays.

However, compared with the de-excitation of L_3_ vacancies, that of L_2_ has an additional effective channel, L_2_–L_3_X CK transition, besides the X-ray emission and Auger transition. Due to the absence of M-, N- and O-shell electrons in the multiple ionization, part of the CK transitions are suppressed, and the Lβ_1_ X-ray emission will be enhanced correspondingly. In addition, there are three channels for the de-excitation of L_1_ vacancies, which are X-ray emission, Auger transition and CK transition. The multiple ionization of M-. N- and O-shell will weakens the process of non-radiation transition, and lead to the enhancement of Lβ_3, 4_ X-ray emissions. As a whole result, the relative intensity ratios of Lβ_1, 3, 4_ to Lα_1, 2_ X rays are enlarged, as shown in Fig. 3. This ratio is essentially constant with the incident energy. This further explains that the multiple ionization degree of outer shells is basically invariant with the projectile energy in the present work.

Lβ_2,15_ and L_α1,2_ X rays mainly come from the transition of N and M electrons to the same lower energy level L_3_, respectively. There are mainly two channels for the de-excitation of L_3_ vacancies, Auger transition and X-ray emission. The sum of fluorescence yield ω_3_ and Auger yield a_3_ is unity: ω_3_ + a_3_ = 1. When multiple electrons are absent in the shells such as M and N, the Auger transition filling the L_3_ vacancies is decreased, and correspondingly, the X-ray emission is enhanced. For iodine, a_3_ is about 2–3 orders of magnitude larger than ω_3_ of L_3_-subshell X rays. The probability of M_5, 4_–L_3_ radiation transition is about 6 times as large as that of N_5, 4_–L_3_ transition^47,48^. Therefore, the fluorescence yield of Lβ_2,15_ X ray is more susceptible to multiple ionization. This will cause the relative intensity ratio of Lβ_2,15_ to Lα_1,2_ X ray to be greater than the atomic data. The extent of multiple ionization is almost independent of the incident energy. So, the ratio is almost constant with the increasing incident energy, as shown in Fig. 4.

In the same way, the ratio of I(Lι)/I(Lα_1, 2_) can be understood easily. The fluorescence yield of Lι X ray is about 1/30 of that of Lα_1, 2_ X ray^47,48^. Therefore, the enhancement of Lι X-ray emission produced by the multiple ionization of M-, N- and O-shell is greater than that of Lα_1, 2_ X ray. As shown in Fig. 5, the relative intensity ratios of Lι to Lα_1, 2_ X ray are higher than the theoretical data for single ionization atom, and have no significant change with incident energy duo to the independent of multiple ionization on projectile energy in the present work.

Theoretically, the fluorescence yield of the single ionized atomic N_4, 5_–L_3_ transition is about 5 times larger than that of the M_1_–L_3_ transition. Compared to atomic data, the experimental enhancement of Lι X-ray emission will be larger than that of Lβ_2, 15_ X ray. As shown in Fig. 6, the experimental ratio of I(Lι)/I(Lβ_2, 15_) is about 1.5 times higher than the theoretical simulation. This also can be deduced from the results in Figs. 5 and 6, where it can be found that the experimental ratio of I(Lβ_2, 15_)/I(Lα_1, 2_) is about 1.5 times of the theoretical value, and that is about 2.2 for the ratio of I(Lι)/I(Lα_1, 2_). This is further verified that, the smaller the probability of radiation transition, the greater the change in fluorescence yield affected by the multiple ionization of the out shell, and the greater the enhancement magnitude of the corresponding X-ray emission.

Figure 7 presents the relative intensity ratio of Lγ_2, 3, 4, 4_' to Lγ_1_ X ray. It is clear that the measured value is larger than the theoretical data of single ionized atom, and the ratios are almost identical with increasing incident energy. This can be interpreted by analogy with the results in Fig. 3. Lγ_1_ X ray comes from the radiation transition of N_4_-L_2_. Lγ_2, 3, 4, 4_' X ray mainly consists of four lines for the radiation transitions of N_3_/N_2_/O_3_/O_2_-L_1_. The fluorescence yield of those two X rays is 0.0028 and 0.0004, and the Auger yield a_2_ and a_1_ at the corresponding lower energy levels L_2_ and L_1_are 0.767 and 0.495, respectively^47,48^. The probability of Auger transition is about 2 to 3 orders of magnitude higher than that of radiation transition. This will result in a significant enhancement of the X-ray emission, under the influence of multiple ionization. The fluorescence yield of Lγ_2, 3, 4, 4_' X ray is about one order of magnitude smaller than that of Lγ_1_ X ray. Therefore, Lγ_2, 3, 4, 4_' X-ray emission is more susceptible to multiple ionization. As a result, the ratio of I(Lγ_2, 3, 4, 4_')/I(Lγ_1_) will be enlarged. In addition, there has one more channel of L_1_-L_2_Y CK transition for the filling of L_1_ vacancies than that of L_2_ subshell. This will also lead to an increase in the fluorescence yield ω_1_ for the de-excitation of L_1_ vacancies. Taking the above two points into consideration, when the outer shells are multiply ionized. The fluorescence yield of Lγ_2, 3, 4, 4_' X ray has a greater increase than that of Lγ_1_. As a result, the experimental ratio of I(Lγ_2, 3, 4, 4_')/I(Lγ_1_) is larger than the theoretical value.

In summary, compared to the theoretical calculation, the experimental relative intensity ratio of the L-subshell X ray is enlarged and goes over 1.2–2.2 times the theory prediction, as shown in Figures 3, 4, 5 and 7. This provides another evidence for the multiple ionization state of the projectile. In the present experimental energy region, the multiple ionization degree is basically constant as the incident energy increases. With the multiple ionization in the M-, N- and O- shells, the fluorescence yield of L-shell X ray is enlarged, because the radiationless transition process is suppressed duo to the absence of some M-, N- and O-shell electrons. The smaller the fluorescence yield of the single ionized atom, the greater the change in the multiple ionization fluorescence yield. As a result, the observed relative intensity ratios are larger than the theory values. Each ratio is different owing to the various changes in fluorescence yield, but it is basically invariant with the change of incident energy.

## Conclusions

I L-shell X-ray emission for 2.5–5.0 MeV I^22+^ ions impacting on Fe target was measured. The energy and the relative intensity ratio of the subshell X rays have been investigated as function of the incident energy. The results indicate that, in the energy region near the Bohr velocity, the projectiles undergo simultaneously the dual effects of ionization and electron capture during the interaction with target atoms below the surface. In addition to the neutralization, the I^22+^ ions are also ionized. Not only the L-shells are ionized, but also the outer-shells, such as M-, N- and O- shells, are multiply ionized. At the balance of ionization and electron capture, the M-, N- and O- shells remain multiple ionization states when the L-shell X-ray is emitted. This leads to a blue shift of the X-ray energy and an increase in the fluorescence yield. Moreover, the smaller the atomic fluorescence yield, the larger the increase in fluorescence yield caused by multiple ionization.
